# Insights into the composition and assembly mechanism of microbial communities on intertidal microsand grains

**DOI:** 10.3389/fmicb.2023.1308767

**Published:** 2023-11-30

**Authors:** Meng Wang, Kun Zhao, Xuan Li, Bin-Bin Xie

**Affiliations:** State Key Laboratory of Microbial Technology, Institute of Microbial Technology, Shandong University, Qingdao, China

**Keywords:** marine sand grains, microscale, heterogeneity, stochastic process, microbial interaction

## Abstract

**Introduction:**

Marine microorganisms are essential in marine ecosystems and have always been of interest. Currently, most marine microbial communities are studied at the bulk scale (millimeters to centimeters), and the composition, function and underlying assembly mechanism of microbial communities at the microscale (sub-100 micrometers) are unclear.

**Methods:**

The microbial communities on microsand grains (40–100 µm, *n* = 150) from marine sediment were investigated and compared with those on macrosand grains (400–1000 µm, *n* = 60) and bulk sediments (*n* = 5) using amplicon sequencing technology.

**Results:**

The results revealed a significant difference between microsand grains and macrosand grains. Microsand grains had lower numbers of operational taxonomic units (OTUs_(97%)_) and predicted functional genes than macrosand grains and bulk-scale samples. Microsand grains also showed greater intersample differences in the community composition and predicted functional genes than macrosand grains, suggesting a high level of heterogeneity of microbial communities at the microscale. Analyses based on ecological models indicated that stochastic processes dominated the assembly of microbial communities on sand grains. Consistently, cooccurrence network analyses showed that most microbial cooccurrence associations on sand grains were highly unstable. Metagenomic sequencing and further genome-scale metabolic modeling revealed that only a small number (1.3%) of microbe pairs showed high cooperative potential.

**Discussion:**

This study explored the microbial community of marine sediments at the sub-100 µm scale, broadening the knowledge of the structure and assembly mechanism of marine microbial communities.

## Introduction

The organization of microbial communities influences various properties, such as metabolism, community stability, and intermicrobial interactions (Coyte et al., 2015; Nagara et al., 2017; Sheth et al., 2019). However, currently used methods for microbiome studies often result in the loss of microbial organization information (Sheth et al., 2019). For example, most complex microbial communities exhibit significant microscale (micrometres) heterogeneity (Kuroda et al., 2016; Leventhal et al., 2018; Armitage and Jones, 2019), and traditional bulk-scale (millimeters to centimeters) samples could contain thousands of subcommunities and provide only average information on these subcommunities (Cordero and Datta, 2016; Shi et al., 2020). In addition, current microbial community profiling methods, such as metagenomic sequencing, require homogenized material (Sheth et al., 2019). All these factors will lead to the loss of spatial information. Therefore, the distribution information on microbial communities at the microscale in diverse environments is poorly understood, although a few samples from environments such as the intestinal tract, oral cavity and activated sludge have been studied (Sheth et al., 2019; Shi et al., 2020; Chen et al., 2022; Zhao et al., 2023). Approximately 71% of the Earth’s surface is occupied by oceans, but knowledge of the microscale microbial distribution in marine environments is limited to the 17 sand grains (grain size approximately 1 mm) reported by Probandt et al. (2018), and the distribution of marine microorganisms at the microscale (especially for the scale <100 μm) has not yet been studied.

Microbial interactions occur at the microscale (~100 μm) in most complex microbial communities (Gantner et al., 2006; Cordero and Datta, 2016), and various network models have been widely used to infer interactions between microorganisms (Faust and Raes, 2012; Yuan et al., 2021). Currently, most microbial community information is derived from bulk-scale samples. Thus, it is worth noting that the mismatch between the spatial scales of species interactions and physical dimensions of typical microbial community samples may lead to erroneous conclusions about population parameters and species interactions (Armitage and Jones, 2019). Studies at very small scales will yield more reliable inferences about ecological mechanisms structuring microbial communities (Cordero and Datta, 2016; Armitage and Jones, 2019).

The microbial community assembly mechanism is a central issue in microbial ecology. The neutral-based theory suggests that stochastic processes, such as birth, death and speciation, shape biological communities (Chave, 2004; Ning et al., 2020). Conversely, the niche-based theory suggests that biological communities are regulated by deterministic abiotic and biotic factors, including environmental factors and species interactions (Chave, 2004; Ning et al., 2020). Recently, several studies have revealed the assembly mechanisms of marine microbial communities and found that the mechanisms varied across different marine environments (Allen et al., 2020; Zhang et al., 2022). Since these studies were conducted using bulk-scale samples, the microbial community assembly mechanism at the microscale remains unclear. Microbial communities in the intertidal zone are affected by water flow (Zhang et al., 2022), and the environmental factors (e.g., temperature and oxygen content) of sand grains at different depths may differ (Zhou et al., 2013), while the exchange of metabolites between microorganisms is convenient at the microscale (Cordero and Datta, 2016). These factors may affect the assembly of microbial communities at the microscale, making it interesting to study the assembly mechanism of microbial communities on sand grains.

In this study, the microbial communities of intertidal surface sediment bulk-scale samples (1–2 cm), macrosand grains (400–1,000 μm), and microsand grains (40–100 μm) were studied using amplicon sequencing technology and metagenomic sequencing technology. The microbial community compositions and predicted functions were compared between samples. Ecological models were employed to investigate the assembly mechanism of the community. Cooccurrence network analyses and the genome-scale metabolic modeling-based approach were used to study the cooperative potential among microbes. The results of this study will expand the understanding of the distribution and assembly of microbial communities on marine sand grains.

## Materials and methods

### Sample collection

**Figure 1:**
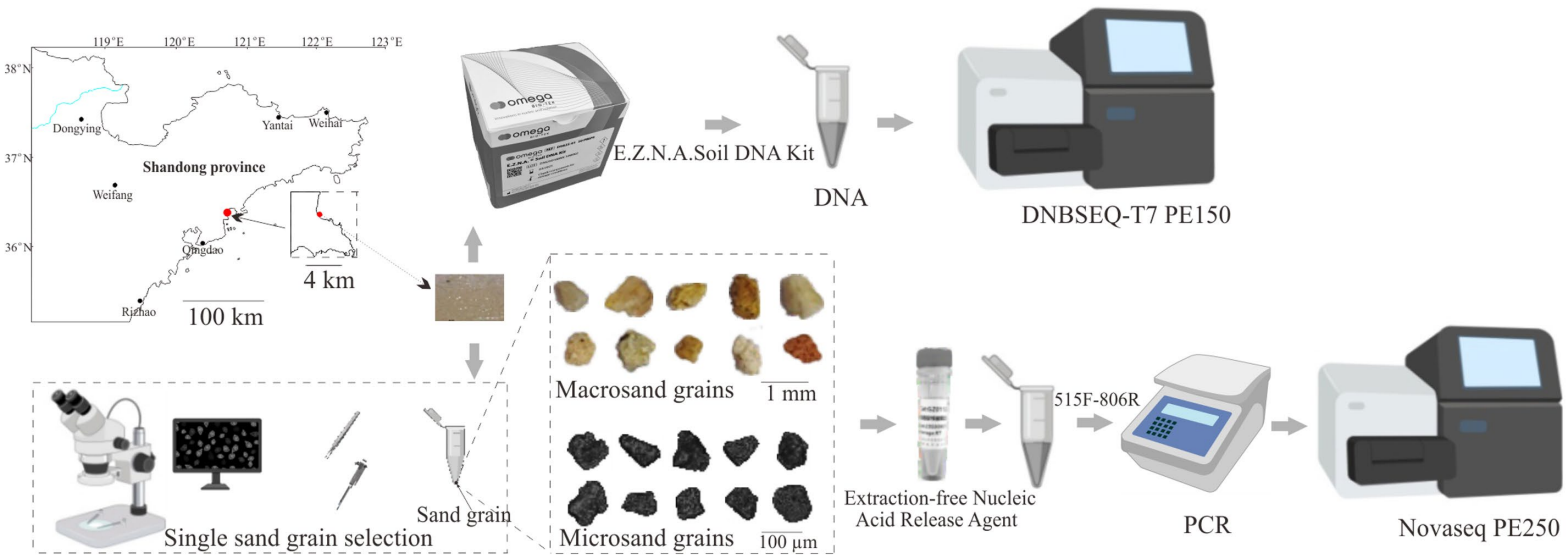
Schematic graph showing the experimental workflow of this study.

The intertidal surface sediment samples were collected at Qingdao Binhai Park, Shandong Province (36°22’ N, 120°41′ E) in May 2023 using sterilized stainless steel spoons (Figure 1). In order to maintain the integrity of the sample and avoid the sample being dispersed by seawater, sediment samples were collected from the intertidal zone 1 h prior to flooding by seawater. The sampling procedure was as follows. First, we delineated a 5 cm × 5 cm nearly square sampling area in the intertidal zone using a stainless steel ruler. Then, five 1 cm × 1 cm sampling zones were located at the four corners and the center of the square sampling area, and the samples collected from 1 cm × 1 cm sampling zones were regarded as bulk-scale samples. Finally, the remaining samples within the 5 cm × 5 cm square sampling area were collected for subsequent sorting. The sampling depth was in the range of 0–2 cm. The collected samples were stored in 50 mL sterilized centrifuge tubes and transported to the laboratory for processing within 20 min.

### Single sand grain separation, DNA extraction, PCR, and Illumina sequencing

For bulk-scale samples, a total of five independent DNA extractions from each 0.2 g of intertidal surface sediment samples were performed. Total genomic DNA was extracted using the E.Z.N.A Soil DNA Kit (OMEGA, Norcross, Georgia United States) according to the manufacturer’s protocol (Figure 1).

To obtain macrosand grains (400–1,000 μm), intertidal surface sediment samples were resuspended in PBS and passed through 1,000 and 400 μm sterilized nylon mesh. Single macrosand grain was randomly hand-sorted using sterilized forceps on a super clean bench, and single macrosand grain was placed in a sterile PCR tube. A total of 75 macrosand grains were collected. To obtain microsand grains (40–100 μm), the sand grains that passed through 400 micron nylon were subjected to 100 μm (BIOLOGIX) and 40 μm cell strainers (BIOLOGIX) (Chen et al., 2022). For each size cell strainer, sand grains were passed through three times. Then, the microsand grains were resuspended in 50% ethanol in PBS and subjected to hand sorting to obtain single sand grains (Chen et al., 2022). Single microsand grains were randomly hand-sorted using a 2.5 μL pipette under a high-definition video microscope (GP-660 V, Kunshan Gaopin Precision Instrument Co. LTD.) on a super clean bench, and each single sand grain was then transferred into a sterile PCR tube. A total of 180 microsand grains were collected. The Extraction-free Nucleic Acid Release Agent (GZ011201, Chenyi Jingze Biotechnology Co. Ltd., Qidong, China) was used to extract DNA from single sand grains according to the manufacturer’s protocol. Briefly, 5 μL of nucleic acid release agent was added to the PCR tube containing the sand grain and centrifuged for 30 s (S1010E, Scilogex) to immerse the sand grain in the release agent, incubated at 95°C for 15 min, mixed with shaking, and then the lysate was taken for PCR amplification (Figure 1). Blank controls without sand grain were set up in all assays.

Universal bacterial primers 515F: GTG YCA GCM GCC GCG GTA A and 806R: GGA CTA CNV GGG TWT CTA AT were used to amplify the V4 region of the 16S rRNA gene of the genomic DNA (Apprill et al., 2015; Parada et al., 2016). For the bulk samples, PCR amplification was performed in a total volume of 50 μL containing 1 μL bovine serum albumin (20 mg ml^−1^), 25 μL of 2 × Taq Plus Master Mix (P212, Vazyme, China), 1 μL of forward primer (1 μM), 1 μL of reverse primer (1 μM), 1 μL of DNA template (10 ng/μl) and 21 μL nuclease-free water. For the single sand grain, PCR amplification was performed in a total volume of 50 μL containing 1 μL bovine serum albumin (20 mg ml^−1^), 25 μL of 2 × Taq Plus Master Mix (P212, Vazyme, China), 1 μL of forward primer (1 μM), 1 μL of reverse primer (1 μM), 4 μL of sample lysate and 18 μL nuclease-free water. The thermocycling program was as follows: 98°C for 2 min; 25 cycles of 98°C for 10 s, 55°C for 30 s, and 72°C for 40 s; 72°C for 5 min; and 10°C hold. Amplification products were purified using a DNA Clean-up Kit (CW2301M, CWBIO, China), and agarose gel electrophoresis showed no bands in the blank controls. Then, the products underwent a short amplification with Illumina adapters (98°C 30 s, 8 cycles of 98°C 10 s, 55°C 30 s, 72°C 40 s, 72°C 5 min, 10°C hold). The PCR products were purified and then sequenced using the Illumina NovaSeq 6,000 platform, yielding 250 bp paired-end reads (Figure 1).

### Bioinformatics

Low-quality sequences in raw data were filtered out using Fastp (v 0.23.2) (Chen et al., 2018), and the paired sequences were merged using Usearch (v 11.0.667). Only sand grains that obtained more than 10,000 sequences were reserved for subsequent analysis. Sequences with ≥97% similarity were assigned to the same OTU_(97%)_, and the chimeric sequences were removed using UCHIME (Edgar et al., 2011). OTU_(97%)_ taxonomy annotation was performed with the Silva_123 database as a reference database using Usearch (v 11.0.667). The Shannon index and Bray–Curtis distances were calculated using the vegan package in R software (v 4.3.1). Permutational multivariate analysis of variance (PERMANOVA) was performed to test the significance of differences in the microbial community using the vegan package in R software (v 4.3.1). In this study, the coefficient of variation was defined as the standard deviation divided by the mean and was used to reflect the variation degree in the relative abundance of a certain taxon between sand grains (Chen et al., 2022; Zhao et al., 2023). To determine pairwise associations between OTUs_(97%)_, OTUs_(97%)_ with relative abundances greater than 0.1% in at least 10% of sand grains were selected for the cooccurrence analysis. A significant association between two OTUs_(97%)_ was determined if the Spearman’s correlation coefficient was <−0.4 or >0.4 and the *q*-value was <0.05 (Benjamini–Hochberg). Next, we plotted and analyzed the cooccurrence networks using Gephi (v 0.9.2) (Bastian et al., 2009). Phylogenetic Investigation of Communities by Reconstruction of Unobserved States (PICRUSt2) was used to infer the microbial function of the bulk sample and sand grains (Douglas et al., 2020).

### Analysis of microbial community assembly

To determine the role of determinism and stochasticity in sand grain microbial community assembly, a previously described null model-based analysis was used (Ning et al., 2020). Briefly, the OTUs_(97%)_ were first divided into different bins based on their phylogenetic relationships using the iCAMP (v1.3.4) package in R software (v 4.3.1) (Ning et al., 2020). Then, the beta net relatedness index (βNRI) and β-diversities using the modified Raup-Crick metric (RC) of each bin were calculated. According to the values of the βNRI and RC parameters, the bins could be considered the percentages of five processes: heterogeneous selection (βNTI < −1.96), homogeneous selection (βNTI > 1.96), homogenizing dispersal (|βNTI| < 1.96, RC < −0.95), dispersal limitation (|βNTI| < 1.96, RC > 0.95), and drift (|βNTI| < 1.96, |RC| < 0.95). The relative importance of individual processes at the whole community level was the sum of the fractions of individual processes across all bins weighted by the relative abundance of each bin. The parameter *n*_min_ was 24, and it was determined in an indirect way (pNST) (Ning et al., 2020). In addition, a neutral community model (NCM) was also used to predict the importance of stochastic processes on community assembly using R software (v 4.3.1) (Sloan et al., 2006; Chen et al., 2019). This model is an adaptation of the neutral theory adjusted to large microbial populations, and the parameter *R*^2^ represents the overall fit to the neutral model (Sloan et al., 2006).

### Metagenomic sequencing, assembly, and binning

The genomic DNA of bulk-scale samples was also used for metagenome shotgun sequencing. The DNA library was constructed and sequenced at Personalbio Technology Co., Ltd. (Shanghai, China) using the DNBSEQ-T7 platform. Approximately 500 Gbp (2 × 150 bp paired-end reads) of raw metagenomic data were generated for bulk-scale samples. The raw reads were trimmed with Trimmomatic (v 0.39) (Bolger et al., 2014), and the trimmed sequences were assembled using Megahit (v 1.2.9) with default parameters (Li et al., 2015). Scaffolds longer than 1,000 bp were binned into draft genomes using MetaBAT2 (v 2.15) (Kang et al., 2019). The software RefineM (v 0.1.2) and CheckM (v 1.1.3) were used to obtain the optimized metagenome assembled genomes (MAGs) (Parks et al., 2015, 2017). Only MAGs with integrity (≥50%) and contamination (≤10%) were retained for subsequent analysis. The GTDB-Tk (v 2.1.1) package was used for taxonomic classification of the MAGs (Chaumeil et al., 2020). Prodigal (v 2.6.3) was used to predict the open reading frames (ORFs) of each MAG (−*p* meta) (Hyatt et al., 2010).

### The potential interactions between MAGs

A genome-scale metabolic modeling-based approach described by Du et al. was adopted in this study to explore the potential interactions between microorganisms in intertidal sediments (Du et al., 2022). Briefly, the protein fasta file of each MAG was used to reconstruct the genome-scale metabolic model using CarveMe (v 1.4.1) (Machado et al., 2018), and the gap-filling process was performed with M9 media (Du et al., 2022). The metabolic interaction potential of each pair of unique models was assessed seven times with the same parameters using SMETANA (v 1.0.0) (the global mode) (Zelezniak et al., 2015), given that the results (MIP value) of each SMETANA calculation are not entirely consistent, and finally, the median was taken to represent the cooperation potential of each pair. The MIP scores could reflect the number of essential nutritional components that the pair could provide for each other through metabolic exchange; thus, the MIP scores were used to evaluate the cooperation potential between pairs. Next, according to the criteria described by Du et al. (2022), MIP ≥ 3 was set as the threshold to distinguish the relatively low and high interactions for this community. Briefly, along with the increase in the MIP, the pair number presented an exponential decay that tended to be gentle from MIP = 3, and MIP = 3 was the median value of the MIP interval (0–5) for this community. Thus, MIP = 3 was set as the threshold to distinguish the relatively low and high interactions for this community. SMETANA was also applied to calculate the compounds exchanged in highly interacting pairs (the detailed mode), and only the compounds with a SMETANA score greater than or equal to 0.1 were considered, and inorganic compounds were excluded (Zelezniak et al., 2015; Du et al., 2022).

## Results

### Fewer OTUs_(97%)_ were found on microsand grains than on macrosand grains

**Figure 2:**
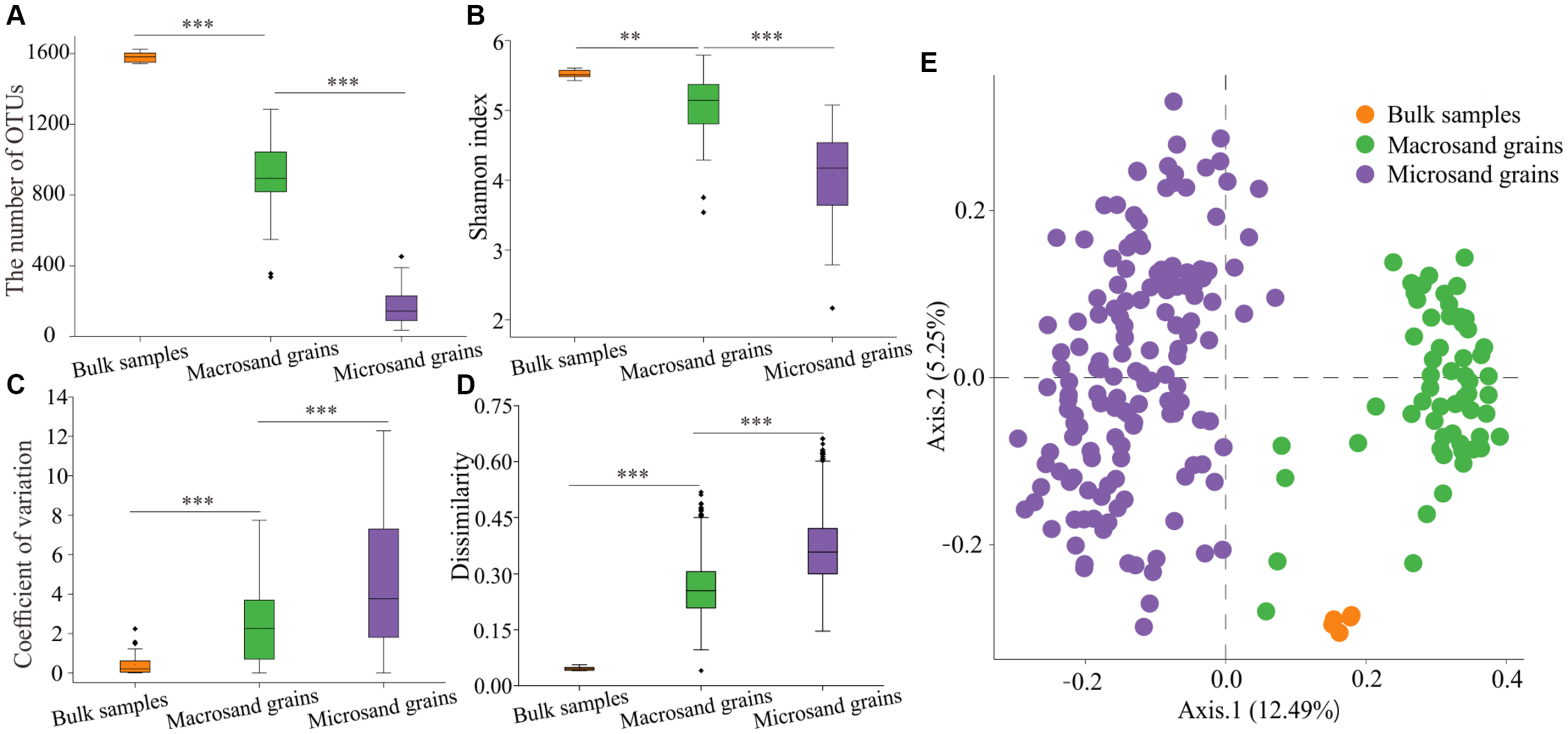
Alpha diversity and beta diversity of microbial communities. **(A)** The OTU_(97%)_ numbers in bulk-scale samples, macrosand grains, and microsand grains. **(B)** The Shannon index in bulk-scale samples, macrosand grains, and microsand grains. **(C)** The coefficient of variation between samples for each OTU_(97%)_. **(D)** Bray–Curtis dissimilarity values between samples of microbial community composition. **(E)** Principal coordinate analysis (PCoA) was used to visualize the communities of bulk-scale samples, macrosand grains, and microsand grains. Significance was determined with the Wilcoxon rank sum test, and * represents *p* < 0.05, ** represents <0.01, and *** represents *p* < 0.001.

In this study, amplicon sequencing technology was used to reveal the microbial community composition in bulk-scale (1–2 cm), macroscale (400–1,000 μm), and microscale (40–100 μm) sediment samples. After removing the low-quality sequences and the samples with sequence numbers less than 10,000, a total of 11,372,765 high-quality 16S rRNA gene sequences were obtained, belonging to 215 samples, including 5 bulk samples, 60 macrosand grain samples, and 150 microsand grain samples. To ensure a fair comparison, we randomly subsampled the sequences to the smallest sample size (*n* = 10,405 sequences) across all samples. The rarefaction curves approached saturation, indicating that the current number of sequences can reflect the microbial community composition of the samples (Supplementary Figure S1). After clustering the sequences, a total of 5,138 OTUs_(97%)_ were obtained. A total of 2,908 OTUs_(97%)_ were found in the bulk-scale sample, of which 2,276 were also present in both macrosand grains and microsand grains (Supplementary Figure S2). The sum of the relative abundance of these 2,276 OTUs_(97%)_ was 96.48% ± 0.28, 92.15% ± 6.50, and 91.45% ± 5.75 in the bulk-scale samples, macrosand grains and microsand grains, respectively (Supplementary Figure S2), suggesting the representativeness of manually picked single macro/microsand grains. Further analyses revealed that, 3 macrosand grains could cover more than 60% OTUs_(97%)_ (relative abundance: 92.58% ± 0.61%) found in the bulk samples (Supplementary Figure S2), and 23 microsand grains could cover more than 60% OTUs_(97%)_ (relative abundance: 91.23% ± 0.24%) found in the bulk samples (Supplementary Figure S2). The total 60 macrosand grains covered 92.67% of OTUs_(97%)_ found in the bulk samples (99.17% in relative abundance) and the total 150 microsand grains covered 85.35% of OTUs_(97%)_ found in the bulk samples (97.24% in relative abundance) (Supplementary Figure S2). As expected, the number of OTUs_(97%)_ harbored in the community decreased significantly with decreasing sand grain size (bulk-scale samples: 1581 ± 35, macrosand grains: 907 ± 181, microsand grains: 169 ± 181) (*p* < 0.001, Wilcoxon test) (Figure 2A), as well as the Shannon index (bulk-scale samples: 5.5 ± 0.1, macrosand grains: 5.1 ± 0.4, microsand grains: 4.1 ± 0.6) (*p* < 0.01, Wilcoxon test) (Figure 2B). The above results suggested that the microbial diversity decreased significantly with decreasing sand grain size.

### Greater intersample difference for microsand grains than macrosand grains

**Figure 3:**
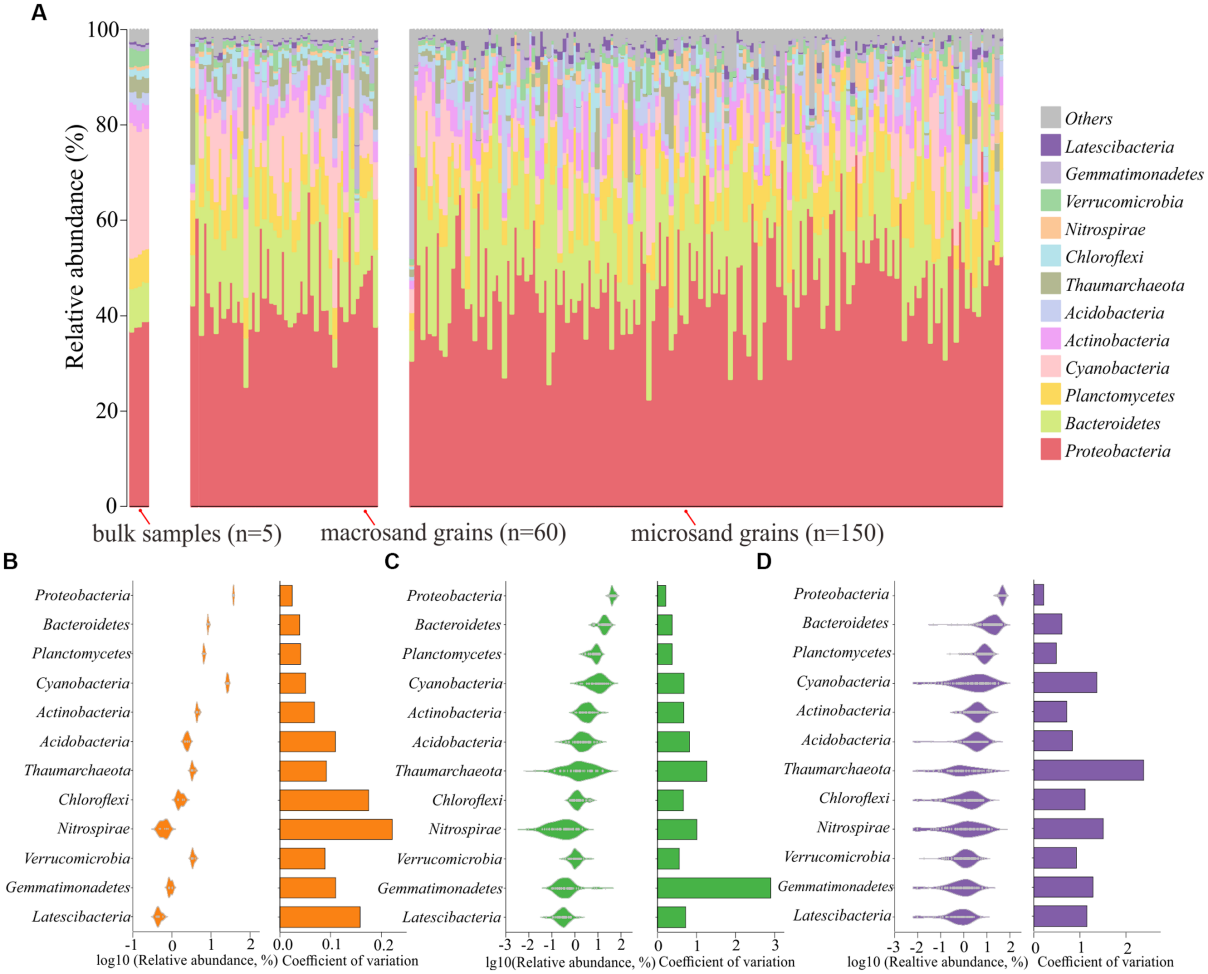
**(A)** Community composition profiles at the phylum level of all samples, including 5 bulk-scale samples, 60 macrosand grains, and 150 microsand grains. Distribution of the dominant phyla across bulk-scale samples **(B)**, macrosand grains **(C)**, microsand grains **(D)**, and the coefficient of variation of relative abundance is shown in the bars on the right.

Further analyses were conducted to reveal the microbial composition on sand grains among different scales. First, the microbial composition of a single sand grain was profiled at the phylum level. As shown in Figure 3A, the microbial community was dominated by *Proteobacteria*, with a mean relative abundance of 46.41%, followed by *Bacteroidetes* (18.47%), *Planctomycetes* (8.02%), and *Cyanobacteria* (7.37%). The community compositions were not uniform among individual sand grains, especially for microsand grains, and evident microscale heterogeneity could be observed. For example, the relative abundance of *Bacteroidetes* fluctuated greatly across microsand grains, ranging from as low as 0.05% to as high as 60% (Figure 3A), and the same was the case for *Proteobacteria* (22.40–74.60%), *Planctomycetes* (0.25–21.40%), *Cyanobacteria* (0–32.3%) and other microbial phyla (Figure 3A). Then, the coefficient of variation for the relative abundance of each phylum was calculated. For the dominant phyla, the coefficients of variation of microsand grains (1.05 ± 0.57) were significantly higher than those of macrosand grains (0.86 ± 0.70) (Wilcoxon test, *p* = 0.038) and bulk-scale samples (0.10 ± 0.06) (Wilcoxon test, *p* = 0.0025) (Figures 3B–D; Supplementary Figure S3).

The microbial composition of single sand grains was also profiled at the OTU_(97%)_ level. The coefficient of variation for each OTU_(97%)_ was also calculated. The results of the coefficient of variation analysis at the OTU_(97%)_ level also supported the conclusion obtained based on the analysis at the phylum level, except that the values of the coefficients of variation at the OTU_(97%)_ level were much higher than those at the phylum level (Figure 2C) (*p* < 0.001, Wilcoxon test). Notably, the distribution of individual OTUs_(97%)_ among the sand grains was extremely heterogeneous. No OTU_(97%)_ was present on all microsand grains. Only 0.59% (25) of OTUs_(97%)_ with high mean relative abundance were found on more than half of the microsand grains, and 42% (1,766) of OTUs_(97%)_ were found only once on microsand grains (Supplementary Figure S4). Furthermore, analyses based on Bray–Curtis distance also showed that the community composition difference between samples of microsand grains was significantly higher than that of macrosand grains and bulk-scale samples (Figure 2D) (*p* < 0.001, Wilcoxon test). Similarly, as shown in Figure 2E, microbial communities from macrosand grains were closer to each other than those from microsand grains. Therefore, all the above results indicated that there was evident heterogeneity in microsand grain microbial communities, and the variation in the microbial communities on the sand grains increased as the sand grain decreased in size.

### Differences in predicted functional gene compositions between microsand and macrosand grains

**Figure 4:**
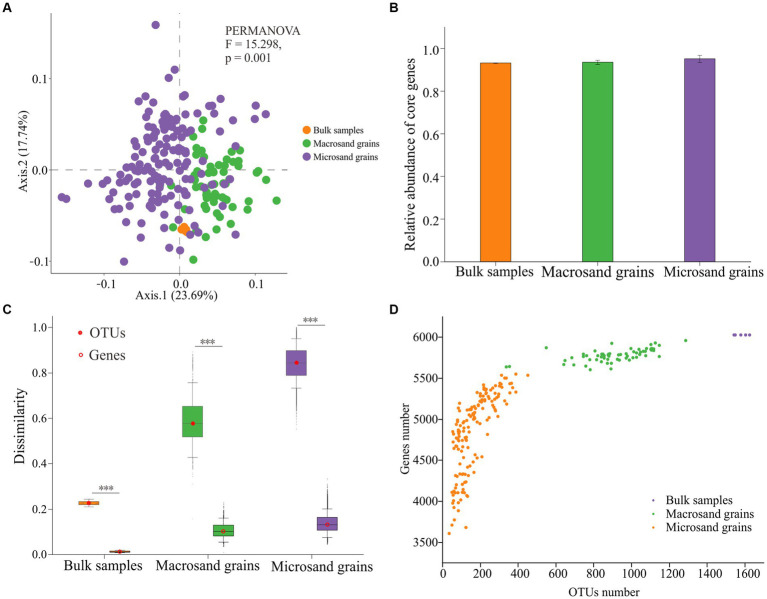
**(A)** Principal coordinate analysis (PCoA) was used to visualize the predicted functional gene compositions of bulk-scale samples, macrosand grains, and microsand grains. **(B)** The mean relative abundance of the 2,510 core functional genes in bulk-scale samples, macrosand grains, and microsand grains. **(C)** Bray–Curtis dissimilarity values of microbial community composition (left) and predicted functional gene composition (right) between samples. Significance was determined with the Wilcoxon rank sum test, and *** represents *p* < 0.001. **(D)** Relationship between the number of OTUs_(97%)_ and the number of predicted functional genes. The horizontal coordinate is the number of OTUs_(97%)_, and the vertical coordinate is the number of functional predicted genes.

A total of 7,190 functional genes were predicted from 215 samples by PICRUSt2 (Supplementary Table S1). There were significant differences in functional gene compositions on sand grains at different scales (Figure 4A). The functional gene composition difference between samples of microsand grains was significantly higher than that of macrosand grains and bulk-scale samples (Supplementary Figure S5) (*p* < 0.001, Wilcoxon test). However, the heterogeneity of functional gene composition was significantly lower than that of microbial composition at all scales (Figure 4C). Notably, unlike the great heterogeneity in the distribution of OTUs_(97%)_ on microsand grains, there were 2,510 shared functional genes (core genes) present on all sand grains (Supplementary Table S1), and the relative abundance of these functional genes exceeded 90% on bulk-scale samples (93.17% ± 0.13), macrosand grains (93.51% ± 1.02), and microsand grains (95.09% ± 1.68) (Figure 4B).

In addition, the relationship between the number of OTUs_(97%)_ and functional genes on sand grains was investigated. As shown in Figure 4D, the slope of the number of functional genes gradually slowed as the number of OTUs_(97%)_ increased. If it was assumed that the functional genes shared on bulk-scale samples were saturated and could complete all ecological functions, microbes on a single macrosand grain could complete nearly all ecological functions (95.85% ± 1.30), and microbes on a single microsand grain could complete most of the ecological functions (80.40% ± 7.96) (Figure 4D; Supplementary Table S1). Furthermore, a total of 351 functional pathways (KEGG level 3) were predicted from OTUs_(97%)_ in the bulk-scale samples (Supplementary Table S2). Macrosand grains contained an average of 345 (98.29%) functional pathways, with three macrosand grains containing all pathways, and microsand grains contained an average of 310 (88.32%) pathways (Supplementary Figure S6).

### Ecological stochastic and deterministic processes shape the sand grain microbial community

**Figure 5:**
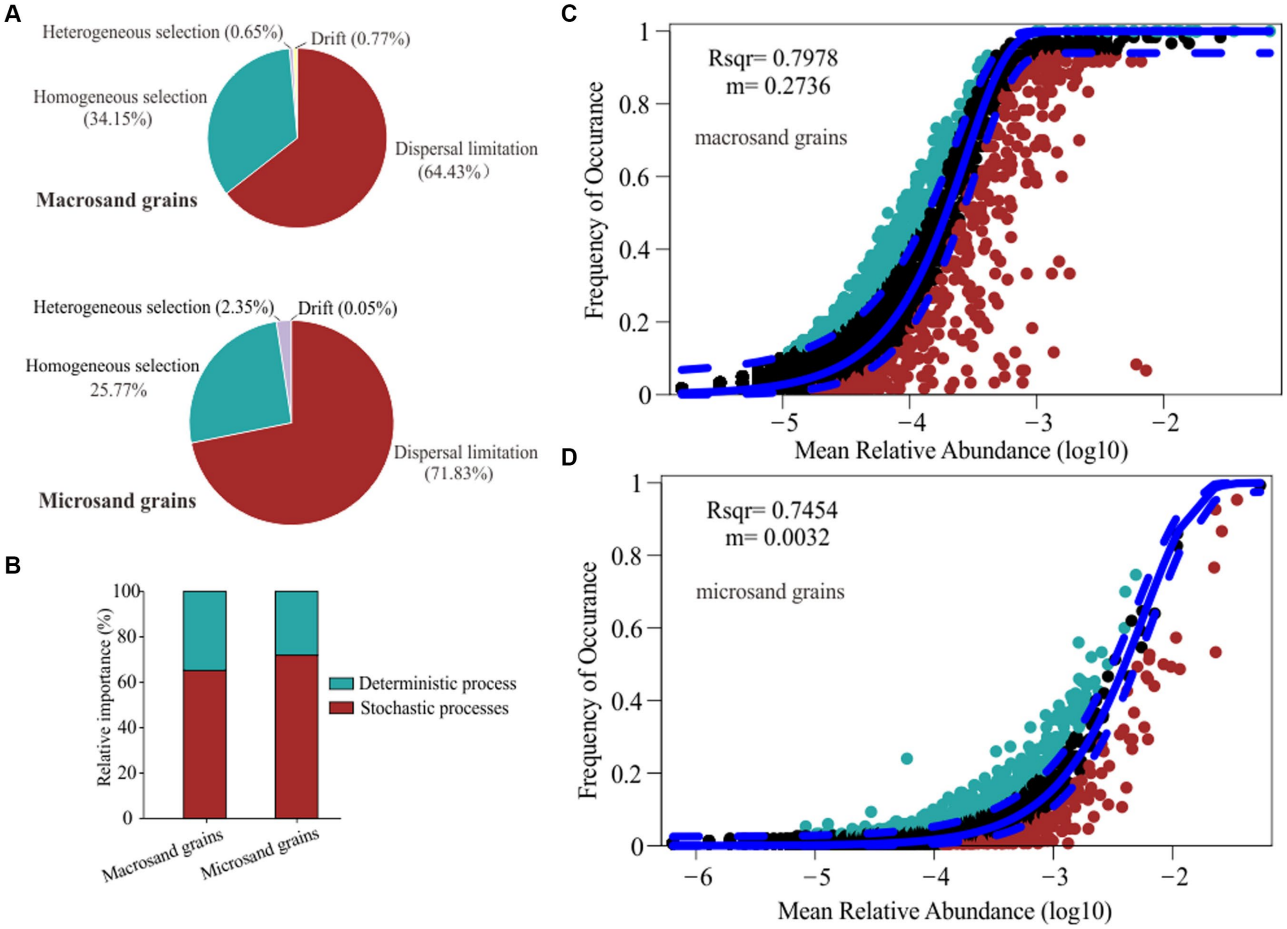
**(A)** The proportion of dispersal limitation, homogeneous selection, heterogeneous selection and drift processes in the microbial assembly process. **(B)** The proportion of stochastic processes and deterministic processes in the microbial assembly process. Fit of the neutral community model (NCM) of macrosand grain microbial community assembly **(C)** and microsand grain microbial community assembly **(D)**. The solid blue lines indicate the best fit to the NCM, and the dashed blue lines represent 95% confidence intervals around the model prediction. OTUs_(97%)_ that occur more or less frequently than predicted by the NCM are shown in different colors. m indicates the immigration rate, and *R*^2^ indicates the fit to this model.

To gain insights into the assembly mechanisms underlying the microbial communities on sand grains, two different ecological models, the null model (icamp.big) and the neutral community model, were used to examine the internal forces driving the assembly of microbial communities on the sand grain. The results based on the null model indicated that stochastic processes (macrosand grains: 65.20%, microsand grains: 71.88%) were more critical than deterministic processes (macrosand grains: 34.80%, microsand grains: 28.12%) (Figure 5B). Dispersal limitation (macrosand grains: 64.43%, microsand grains: 71.83%) and homogeneous selection (macrosand grains: 34.15%, microsand grains: 25.77%) were the two processes that governed the assembly of microbial communities on macrosand and microsand grains (Figure 5A). In addition, the neutral community model successfully estimated most of the relationship between the occurrence frequency of OTUs_(97%)_ and their relative abundance variations (Figures 5C,D), with 79.78 and 74.54% of explained community variance for macrosand grains and microsand grains, respectively. Therefore, this result suggested that stochastic processes were important in shaping the microbial community assembly on sand grains.

### Unstable cooccurrence associations between microorganisms on microsand and macrosand grains

**Figure 6:**
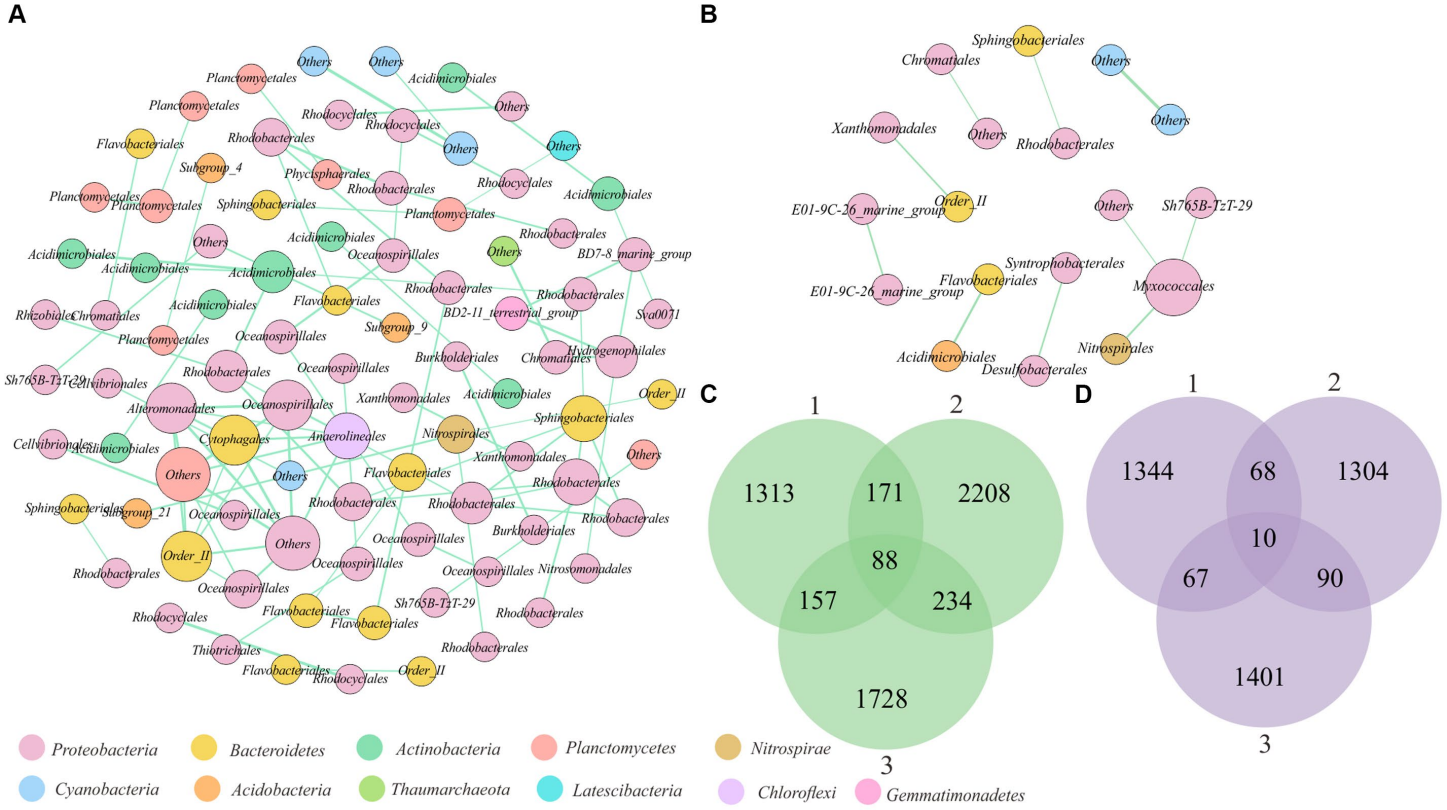
Cooccurrence associations between OTUs_(97%)_ on sand grains. **(A)** Network analysis based on stable cooccurrence associations between OTUs_(97%)_ on macrosand grains. Each node represents an OTU_(97%)_, and the size of the node represents the degree. The green connecting lines represent positive correlations, and the red lines represent negative correlations. **(B)** Network analysis based on stable cooccurrence associations between OTUs_(97%)_ on microsand grains. Each node represents an OTU_(97%)_, and the size of the node represents the degree. The green connecting lines represent positive correlations, and the red lines represent negative correlations. **(C)** Venn diagram of the shared cooccurrence associations between OTUs_(97%)_ of three subsamples of macrosand grains each containing 15, 20, and 25 sand grains. **(D)** Venn diagram of the shared cooccurrence associations between OTUs_(97%)_ of three subsamples of microsand grains, each containing 40, 50, and 60 sand grains.

To investigate whether there were stable associations between microorganisms on sand grains, the cooccurrence patterns within the microbial communities across sand grains were analyzed. To reduce random noise, OTUs_(97%)_ with relative abundances greater than 0.1% in at least 10% of sand grains were selected for cooccurrence network analysis. Finally, a total of 355 OTUs_(97%)_ were used to construct macroscale and microscale cooccurrence networks. To prevent the biases introduced by sampling, co-occurrence networks were constructed repeatedly based on three randomly selected subsamples for macrosand grains (each containing 15, 20, and 25 sand grains) and microsand grains (each containing 40, 50, and 60 sand grains), respectively (Goberna and Verdú, 2022). Notably, the cooccurrence networks constructed by different subsamples varied greatly (Figures 6C,D). Of all the co-occurring pairs inferred from subsamples, only 88 OTU_(97%)_ pairs were stable in the macrosand grains and 10 pairwise OTUs_(97%)_ in the microsand grains (Figures 6C,D). Then, the networks were constructed from the entire set of macrosand grains and microsand grains, respectively, and most of the OTU_(97%)_ pairs consistently found in subsamples were also found in the full samples (macrosand grains: 91%, microsand grains: 100%) (Supplementary Figure S7). Finally, the stable OTU_(97%)_ pairs in macrosand grains and microsand grains were used to construct the cooccurrence network. As shown in Figures 6A,B, all correlations were positive. The OTUs_(97%)_ (network nodes) were generally from phyla such as *Proteobacteria*, *Bacteroidetes*, and *Actinobacteria*, and most associations associated with OTUs_(97%)_ of *Proteobacteria* (macrosand grains: 73%, microsand grains: 80%). Compared to the macrosand grain network, the microsand grain network harbored a simple cooccurrence pattern, and most associations on macrosand grains were absent on microsand grains (Figures 6A,B). Even so, some associations were robust as the spatial scale changed, such as the association between OTU_335 (*Sphingobacteriales*) and OTU_165 (*Rhodobacterales*) and the association between OTU_5383 (*Cyanobacteria*) and OTU_920 (*Cyanobacteria*) (Figures 6A,B). A study of samples from a desalination plant revealed similar distribution patterns for bacteria from the orders *Sphingobacteriales* and *Rhodobacterales* (Al-Ashhab et al., 2022), but the underlying mechanisms are not clear.

### Low proportion of high cooperative potentials between microorganisms in intertidal sediment

**Figure 7:**
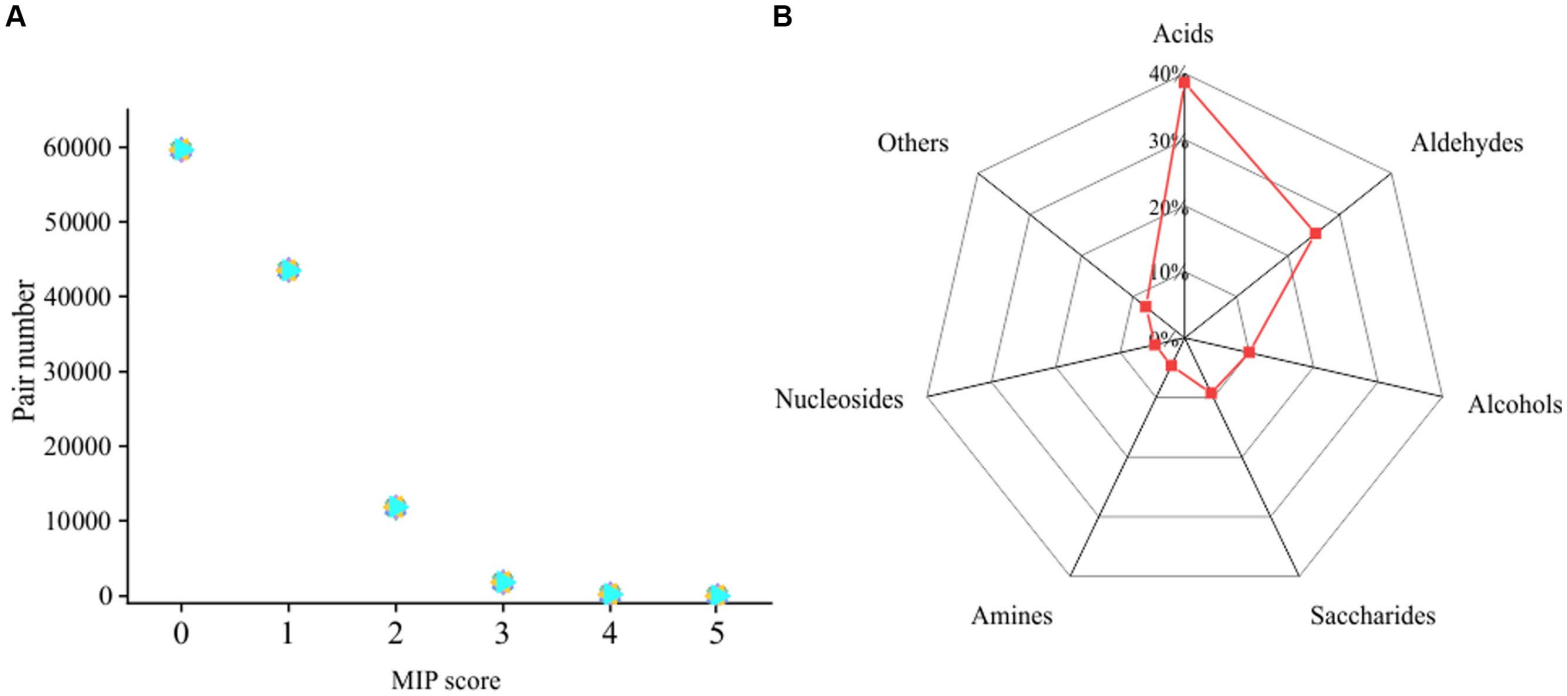
**(A)** Genomic distribution of the MIP scores of all possible pairs of MAGs in the intertidal sediment sample. **(B)** Metabolite classes likely to be exchanged between MAGs predicted by SMETANA.

To understand the cooperative potentials of microorganisms in intertidal sediment, we performed metagenome sequencing of bulk-scale intertidal sediments. After assembly and binning, 484 metagenome assembled genomes (MAGs) (completeness ≥50% and contamination ≤10%) were obtained (Supplementary Table S3). These 484 MAGs were mostly affiliated with 39 phyla, including *Proteobacteria* (122 MAGs), *Chloroflexi* (46 MAGs), *Bacteroidota* (42 MAGs), *Actinobacteria* (37 MAGs) and 237 other MAGs (Supplementary Table S3). Then, a genome-scale metabolic modeling-based approach applied in a previous study was used to infer cooperative potentials between pairwise MAGs (Supplementary Table S4). A total of 116,886 pairs were obtained from 484 MAGs. As shown in Figure 7A, 60,146 (51.5%) pairs showed no cooperation potential (MIP = 0), 55,130 (47.2%) pairs showed low cooperation potentials (MIP = 1, 2), and only 1,610 (1.3%) pairs showed high cooperation potentials (MIP = 3, 4, 5). Therefore, the above results suggested that most of the pairs of microbial individuals did not benefit from each other. A total of 427 MAGs from 37 phyla, including *Proteobacteria*, *Bacteroidota*, and *Myxococcota*, were involved in these 1,610 pairs with high interaction potential, accounting for 88% of the total number of MAGs (Supplementary Tables S3, S4). Furthermore, the exchanged metabolites between high cooperative potential pairs were identified. The amino acids, aldehydes, saccharides and some other compounds were likely exchanged between the pairs with high cooperative potential (Figure 7B).

## Discussion

With the development of metagenomic sequencing technology, the distribution of microbial communities in various environments, such as soils, rivers, and oceans (Chen et al., 2019; Hoshino et al., 2020; Wang et al., 2023), has been well studied. Since most of these studies use bulk-scale samples, we know little about the spatial distribution of microorganisms at the microscale, except for samples in a few environments, such as the human gut, activated sludge, plant rhizosphere and soil aggregates (Sheth et al., 2019; Liu et al., 2021; Chen et al., 2022; Cao et al., 2023). The study of microbial communities at very small scales will lead to more reliable inference of the ecological mechanisms structuring microbial communities (Armitage and Jones, 2019). Knowledge of microbial distribution in marine environments at a small scale has been limited to the 17 sand grains reported by Probandt et al. (2018). They found a highly diverse microbial community in each sand grain, and a core community accounting for >50% of all cells was present on each sand grain (Probandt et al., 2018). In this study, a similar phenomenon was observed on macroscale sand grains. A core community (31 OTUs_(97%)_) with a mean relative abundance of 34% was present on each macrosand grain, which may be a consequence of the size of the macrosand grains in this study being close to that in the study of Probandt et al. (2018). However, regarding microscale sand grains, the situation becomes different. There were no shared OTUs_(97%)_ among microsand grains, which is attributed to the heterogeneity of the microbial community on microsand grains being significantly greater than that on macrosand grains. In addition, an increase in the number of sand grains will also lead to a decrease in the number of shared OTUs_(97%)_. Thus, by comparing microbial communities from different sand grain sizes, this study expanded the knowledge of microbial community assembly on sand grains at small scales. Meanwhile, it is noted that the microbial community of marine sediments changes with seasonal and tidal variations (Zhang et al., 2023; Zhao et al., 2023). Analyses based on time series data in the future will help to obtain a panoramic picture of the assembly of marine microbial communities at the microscale.

The functional heterogeneity among sand grains was significantly lower than the taxonomic composition heterogeneity. The microorganisms on individual microscale sand grains had an average of 80% of the functional genes contained in the bulk-scale samples, even though the number of OTUs_(97%)_ on a single microsand grain was only 11% of the bulk-scale samples. This reflected the functional redundancy in bulk-scale marine sediment samples. Functional redundancy is widespread in the microbial community and reflects the diversity of microorganisms with specific metabolic functions (Louca et al., 2018). This study revealed the distribution of microbial functional genes on a single sand grain, even though this result was obtained by prediction (Douglas et al., 2020). With the development of single-cell sequencing technology, the application of this technology to environmental samples may provide more direct evidence for the distribution of microbial functional genes among sand grains (Lloréns-Rico et al., 2022).

Ecological stochastic and deterministic processes shape the sand grain microbial community, and stochastic processes are more important than deterministic processes. The possible reasons were as follows. First, intertidal surface sediments are strongly affected by tides (Zhang et al., 2022), promoting randomness in microbial community assembly. Several studies have demonstrated that the proportion of stochastic processes increased when the community was disturbed (Dini-Andreote et al., 2015; Zhang et al., 2022), and water flow can affect microbial communities (Zhang et al., 2022). Second, cooccurrence associations between microorganisms in intertidal sediments were mostly unstable, and cooccurrence associations are usually thought to be a result of microbial interactions (Faust and Raes, 2012). Generally, environmental filtering and various biological interactions contribute to deterministic processes (Ning et al., 2020). Our study demonstrated that the biological interactions between microorganisms on sand grains were few and unstable. Finally, *Proteobacteria* was the dominant phylum on sand grains, and the members of *Proteobacteria* usually have broad niches and their community assembly is dominated by random collision and colonization (Wang et al., 2019). Several studies have revealed marine microbial community assembly mechanisms using bulk-scale samples and found that the microbial community assembly mechanisms varied across different marine environments (Allen et al., 2020; Zhang et al., 2022). For example, a study of microbial community assembly processes in marine microplastics revealed that the β-NTI values of all samples were between 0.036 and 0.037, suggesting that stochastic processes dominate microbial community assembly (Zhang et al., 2022). Another study revealed that homogeneous environmental selection dominates microbial community assembly in the oligotrophic South Pacific Gyre (Allen et al., 2020). Here, we found that stochastic processes, especially diffusion limitation, dominated the assembly of microbial communities on sand grains. In this study, we revealed the assembly mechanism of microbial communities on sea sands at the microscale and suggested that stochastic processes are more important than deterministic processes.

Microbial cooccurrence network analysis is often used to describe microbial interactions within communities (Faust and Raes, 2012). The microbial interactions derived from bulk-scale samples tend to reflect the response of abiotic factors such as biogeochemical parameters rather than intermicrobe interactions (Cordero and Datta, 2016), and the study at microscales will yield more reliable inferences (Armitage and Jones, 2019). In this study, the associations between microorganisms on sand grains were highly variable, and only a few co-occurring relationships were stable across different subsample groups (Figures 6C,D). This may be caused by the following reasons. First, most of the microorganisms on the sand grains have no or low cooperation potential with each other (Figure 7A; Supplementary Table S4) (Du et al., 2022), and this low cooperation potential may not be sufficient to support their aggregation. Second, metabolic intermediates can be transported advectively through the sediment matrix in permeable sediments (Probandt et al., 2018). Thus, microorganisms may not need to live together to obtain the required intermediates, resulting in the inability to observe significant spatial cooccurrence relationships. Similarly, Probandt et al. found that some microbial partners occur as densely packed in activated sludge, but on marine sand grains, they were sometimes in close contact with their partner aggregates but often more distant from each other (Probandt et al., 2018).

Though different DNA extraction methods were used for the bulk samples and sand grains, 2,901 of the 2,908 OTUs_(97%)_ found in the bulk samples could be detected in the sand grains, indicating that both methods were efficient. Significant differences in the relative abundance between sand grains and bulk samples were observed for some microbial taxa (*q* < 0.05, Wilcoxon test, BH) (Supplementary Table S5). For example, the relative abundances of *Cyanobacteria* and *Verrucomicrobia* were significantly higher in the bulk samples than in the sand grains, whereas the relative abundances of *Proteobacteria*, *Nitrospirae*, and *Bacteroidetes* were significantly higher in the sand grains than in the bulk samples. Such differences may be explained by different efficiencies of different DNA extraction methods. Other factors may also contribute to the above differences in relative abundance. For example, cells of *Cyanobacteria* often aggregate and some cells may be lost during the filtration pretreatment, resulting in lower relative abundance of *Cyanobacteria* in sand grains than in bulk samples. Interestingly, there were a large number of OTUs_(97%)_ in the sand grains that were not found in the bulk samples, suggesting that analyses based on sand grains may help to recover more community members than that based on bulk samples.

## Data availability statement

The original contributions presented in the study are publicly available. This data can be found at: the Genome Sequence Archive (GSA) of the National Genomics Data Center, China National Center for Bioinformation (CNCB-NGDC) under accession number CRA012803 (https://ngdc.cncb.ac.cn/gsa/). The metagenome-assembled genomes (MAGs) have been submitted to the Figshare database (https://doi.org/10.6084/m9.figshare.24220777.v1).

## Author contributions

MW: Data curation, Formal analysis, Investigation, Visualization, Writing – original draft. KZ: Resources, Validation, Writing – review & editing. XL: Software, Writing – review & editing. B-BX: Conceptualization, Funding acquisition, Project administration, Writing – review & editing.
