# Supplementary material for: Insights into the composition and assembly mechanism of microbial communities on intertidal microsand grains
Source: Front Microbiol. 2023 Nov 30;14:1308767. doi: 10.3389/fmicb.2023.1308767 (PMC10719935; doi:10.3389/fmicb.2023.1308767)
Supplement: Supplementary file 6 [file Data_Sheet_1.docx]

Supplementary Material


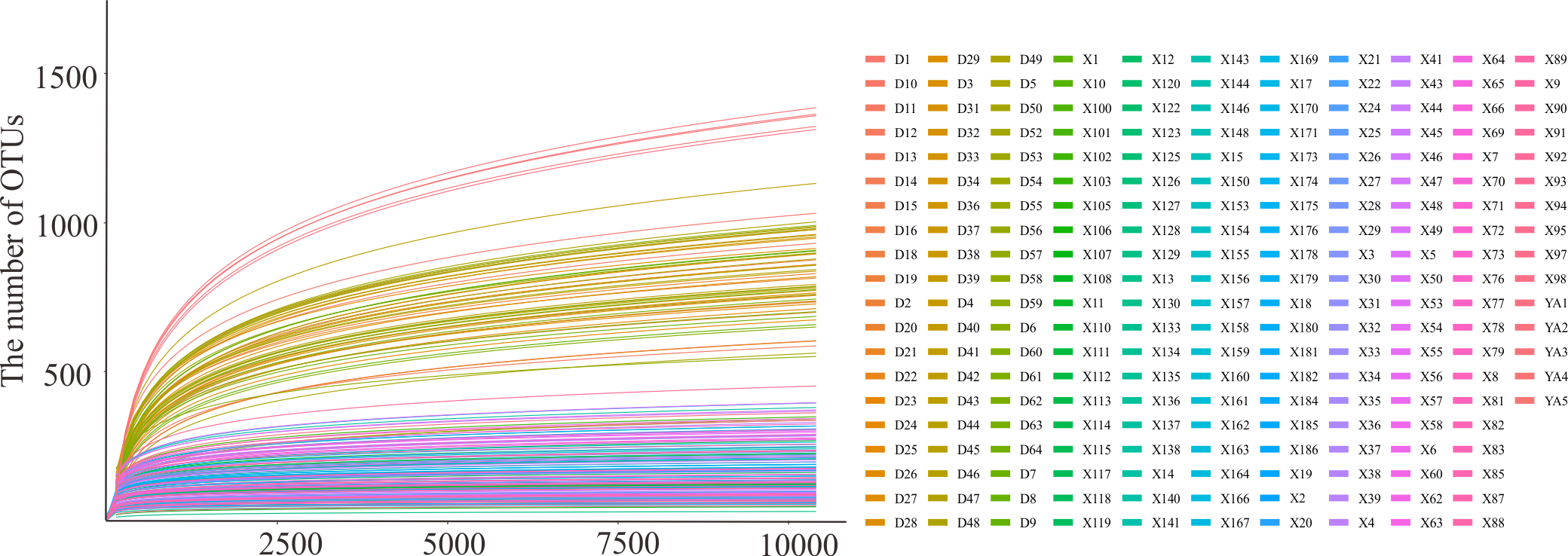


**Supplementary Figure S1.** Rarefaction curve of the 215 samples studied, including 5 bulk sediments, 60 macrosand grains and 150 microsand grains.


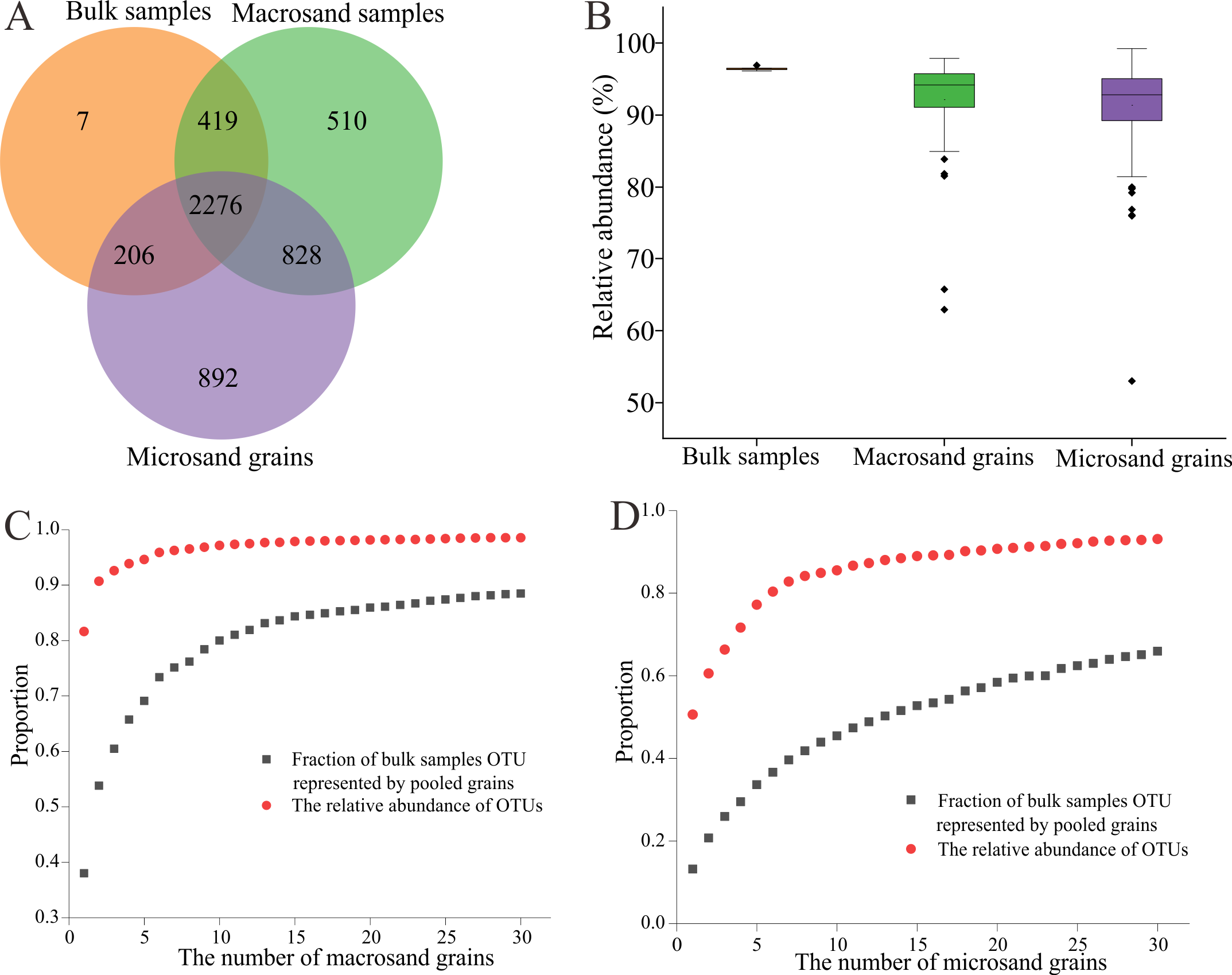


**Supplementary Figure S2.** (A) Venn diagram. The shared OTUs among bulk sediments, macrosand grains and microsand grains. (B) The sum relative abundance of these 2276 shared OTUs in bulk-scale samples, macrosand grains and microsand grains. Fraction of bulk sediment OTU _(97%)_ richness shared by data sets from pools of sand grains (C: macrosand grains; D: microsand grains). Depicted values based on consecutively pooled sand grains in decreasing order of their individual OTU _(97%)_ richness. The 30 sand grains in each group containing the highest number of OTUs _(97%)_ were selected.


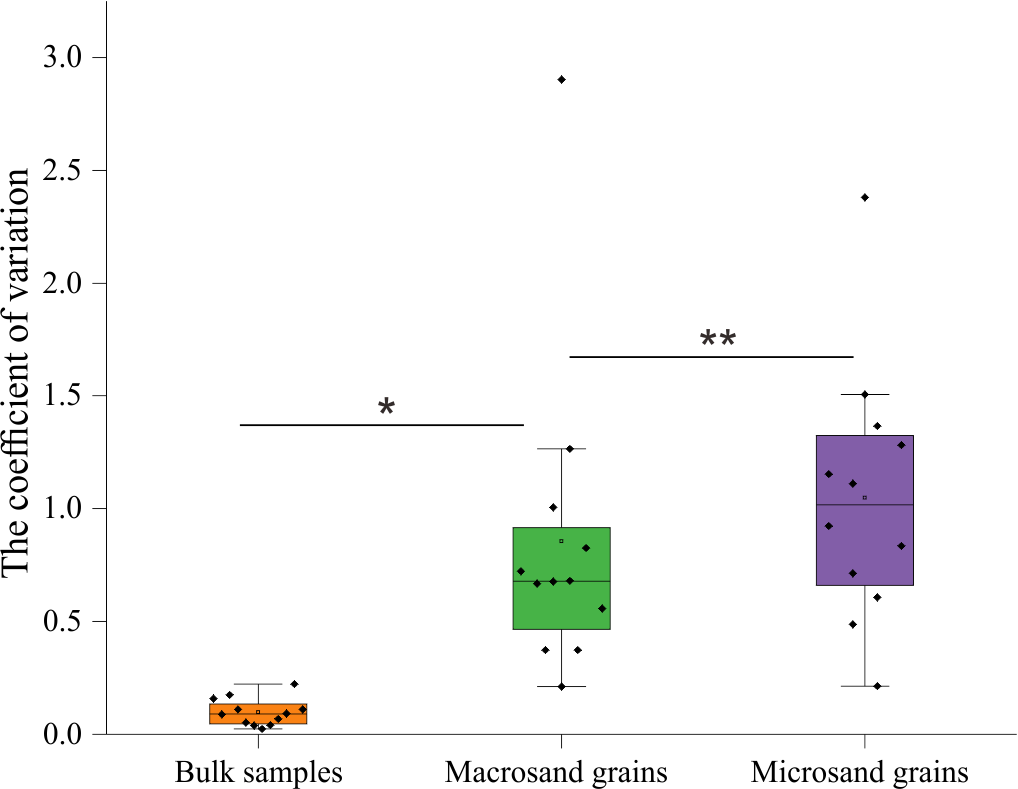


**Supplementary Figure S3.** The coefficient of variation between samples for dominant phyla. Significance was determined with the Wilcoxon signed-rank test, and * represents p< 0.05 and ** represents < 0.01.


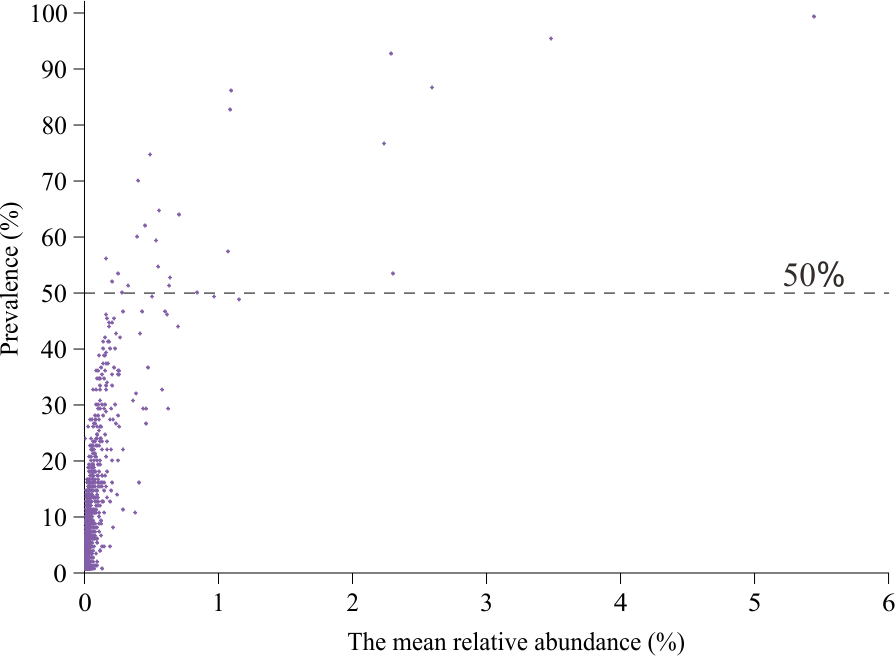


**Supplementary Figure S4.** Relationship between the prevalence of OTUs and their relative abundance. The horizontal axis represents relative abundance, and the vertical axis represents prevalence.


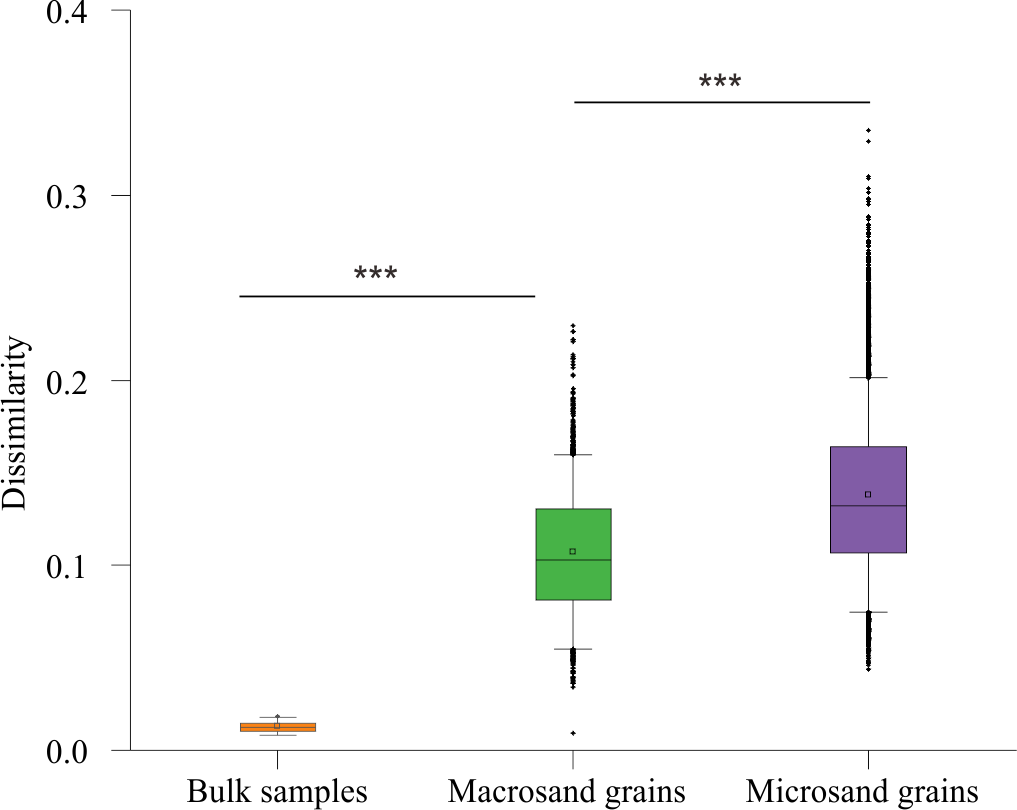


**Supplementary Figure S5.** Bray‒Curtis dissimilarity values of predicted functional gene composition between samples. Significance was determined with the Wilcoxon rank sum test, and *** represents p < 0.001.


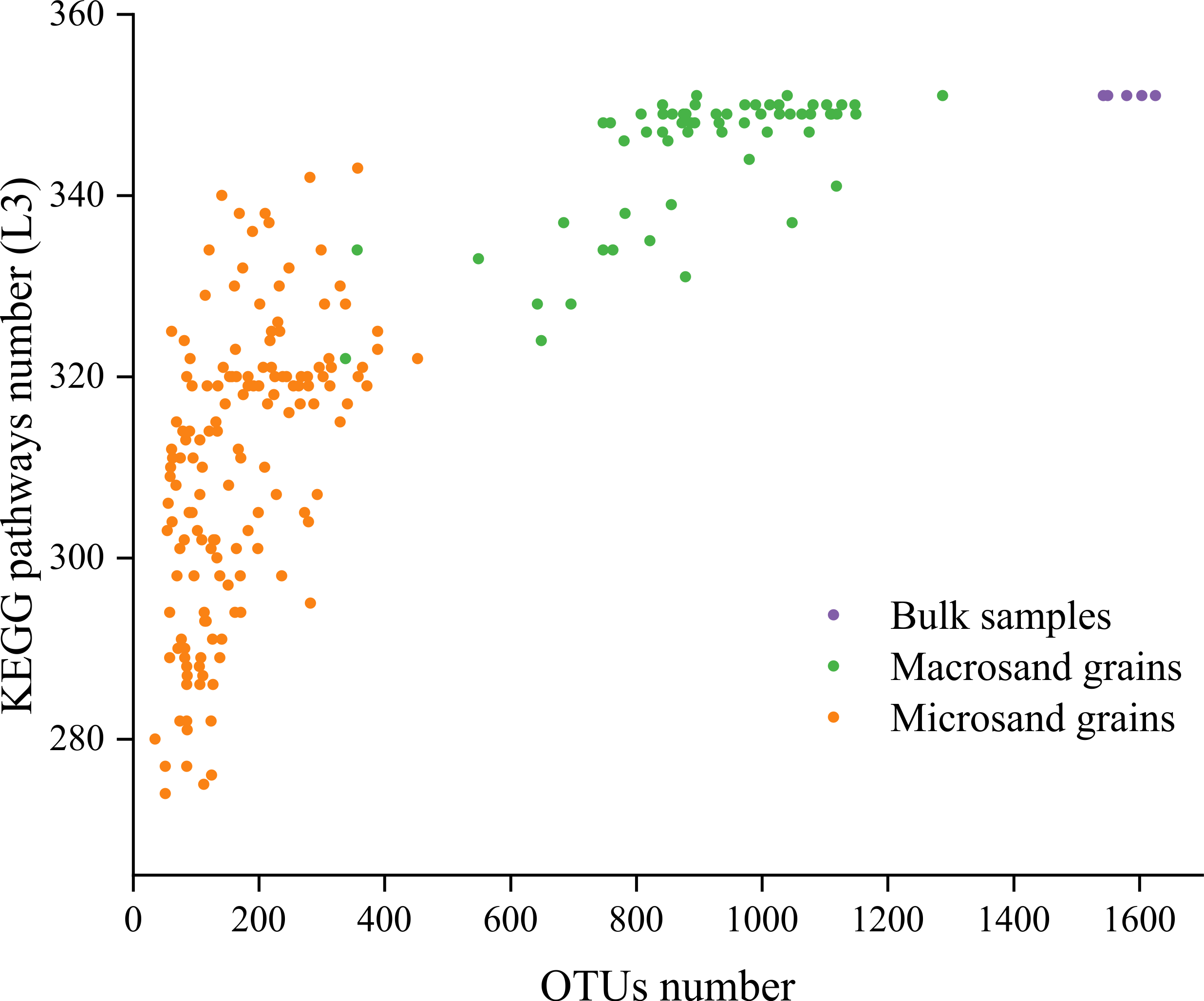


**Supplementary Figure S6.** Relationship between the number of OTUs and the number of KEGG pathways. The horizontal coordinate is the number of OTUs, and the vertical coordinate is the number of KEGG pathways.


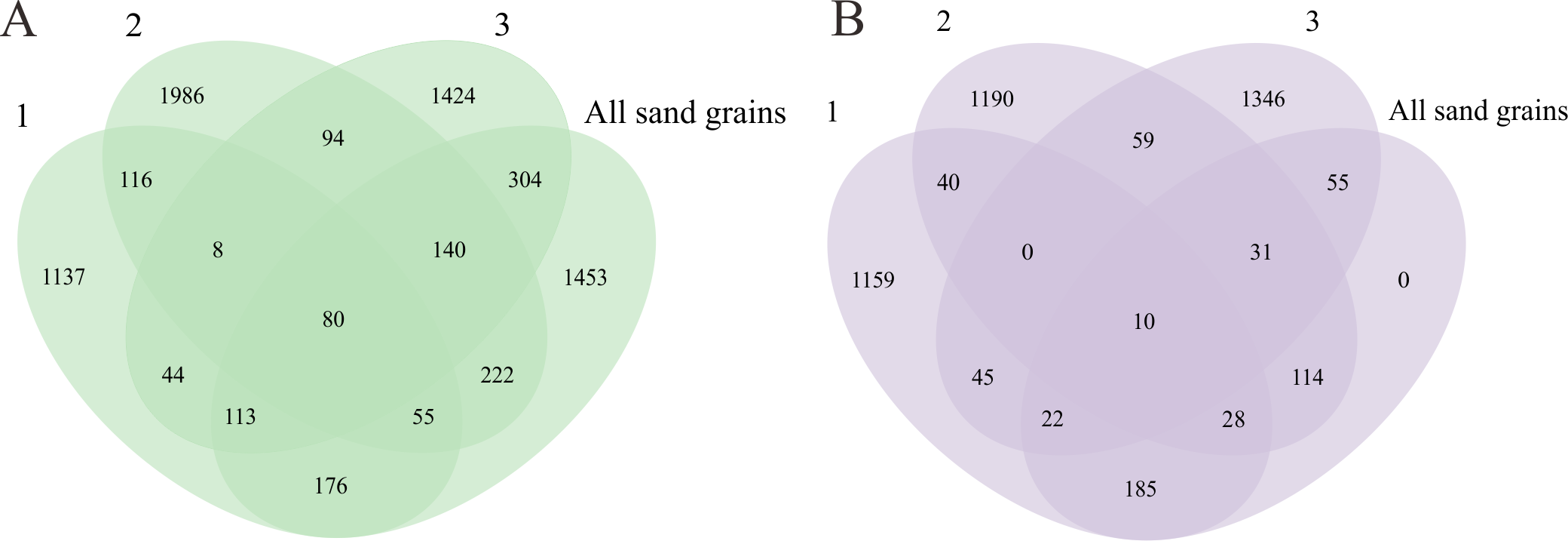


**Supplementary Figure S7.** Venn diagrams of the shared cooccurrence associations between OTUs of three subsamples and the entire set of samples. (A) Macrosand grains. (B) Microsand grains.
